# Telomerase downregulation induces proapoptotic genes expression and initializes breast cancer cells apoptosis followed by DNA fragmentation in a cell type dependent manner

**DOI:** 10.1007/s11033-013-2600-9

**Published:** 2013-05-16

**Authors:** Blazej Rubis, Hanna Holysz, Marta Gladych, Ewa Toton, Anna Paszel, Natalia Lisiak, Mariusz Kaczmarek, Johann Hofmann, Maria Rybczynska

**Affiliations:** 1 Department of Clinical Chemistry and Molecular Diagnostics, Poznan University of Medical Sciences, ul. Przybyszewskiego 49, 60-355 Poznan, Poland; 2 Department of Clinical Immunology, Poznan University of Medical Sciences, Rokietnicka 5d, 60-806 Poznan, Poland; 3 Division of Medical Biochemistry, Biocenter, Innsbruck Medical University, Innrain 80-82, 6020 Innsbruck, Austria

**Keywords:** Adenocarcinoma therapy, Breast cancer, Telomerase, Telomere

## Abstract

The aim of the study was to analyze the consequence of silencing genes coding for the key subunits of the telomerase complex, i.e. *TERT*, *TERC* and *TP1* in human breast cancer MCF7 and MDA-MB-231cells. The transfection was performed using Lipofectamine2000 and pooled siRNAs. The cytotoxic and/or antiproliferative effect of siRNA was measured by the SRB assay, the cell cycle was analysed by flow cytometry and DNA fragmentation by TUNEL analysis. Telomerase activity was assessed by TRAP, followed by PAGE and ELISA assays. Telomerase downregulation was also assessed using qPCR in order to estimate the changes in the expression profile of genes engaged in apoptosis. It was revealed that treatment of breast cancer cells with different siRNAs (100 nM) resulted in a cell type and time-dependent effects. The downregulation of telomerase subunits was followed by reduction of telomerase activity down to almost 60 % compared to control cells. However, a significant effect was only observed when the *TERT* subunit was downregulated. Its silencing resulted in a significant (*p* < 0.05) increase of apoptosis (over 10 % in MCF7 and about 5 % in MDA-MB-231 cells, corresponding to the Annexin V assay) and DNA fragmentation (almost 30 % in MCF7 and over 25 % in MDA-MB-231 cells). Interestingly, also several proapoptotic genes were induced after the downregulation of the key telomerase subunit, including *Bax*, *Bik* or *caspase*-*1* and *caspase*-*14*, as well as *NGFR* and *TNFSF10* which were upregulated twice and more.

## Introduction

High telomerase activity in cancer cells is supposed to be responsible for unlimited divisions and postponing cell death and/or senescence. The enzyme has been shown to be active in over 90 % of all cancer types and in almost 100 % of adenocarcinomas [1, 2], while it is generally inactive, or at least significantly much less active in normal cells. Telomerase activity maintains the telomeres that protect the ends of chromosomes from damage and prevent adverse recombination. It was revealed in many studies that regulation of telomerase is a multifactorial process in mammalian cells, involving the expression of genes coding for telomerase subunits, post-translational protein–protein interactions and protein phosphorylation [3, 4]. Numerous proto-oncogenes and tumor suppressor genes are also engaged in this mechanism. The complexity of telomerase control mechanisms is studied in the context of stem cells renewal, tumor development and ageing [5–7]. Due to important roles of telomerase in those processes it is of great interest to identify the enzyme regulators (tumor suppressors or oncogenes, etc.). Since numerous studies have shown a correlation between short telomere length and increased mortality, the telomerase expression/activity control appears to be one of the most crucial factors to improve cancer prevention, therapy and delay of ageing [8–11].

Transactivation of telomerase, essential for cells immortalization, is supposed to be one of the reasons for cell transformation. Many studies described exogenous factors influencing *TERT*, e.g. several viruses are known to be involved in tumorigenesis of infected tissues [2, 12, 13]. Several strategies have been proposed to control telomerase in cancer cells: anti-sense technology against telomerase RNA component (*TR*) and telomerase reverse transcriptase (*TERT*), ribozymes against *TERT*, anti-estrogens, progesterone, vitamin D, retinoic acid, quadruplex stabilizers, telomere and telomerase targeting agents, modulation of interaction with other proteins involved in the regulation of telomerase and telomeres, etc. However, the transcription control of key telomerase subunits seems to play a crucial role in whole complexes activity and cancer cells immortality. One of the most promising strategies seems to be telomerase downregulation via siRNA technology, in particular in breast cancer, which shows high telomerase expression/activity. Thus, we focused on telomerase downregulation and analysis of RNA interference in breast adenocarcinoma cells.

## Materials and methods

### Cell culture

In order to verify the specificity of telomerase downregulation due to different cell type and basal telomerase expression/activity, two different cancer cell lines were cultured, i.e. estrogen-dependent and *p53* wild type (MCF7) and estrogen-independent and *p53* mutant (MDA-MB-231). Both breast cancer cell lines were cultured in DMEM medium supplemented with 10 % fetal bovine serum (PAA, Pasching, Austria). For cytotoxicity tests, cells were cultured in 96-well plates (5,000 MCF7 and 8,000 MDA-MB-231 cells per well). All other experiments were performed in 12-well plates (30,000 and 50,000 cells per well, respectively) in time intervals of 24, 48 and 72 h. Additionally, non-cancer breast cells, MCF10A, were also cultured in DMEM/F-12 (1:1) (Sigma D6559) supplemented with l-glutamine (2 mM), horse serum (5 %), insulin (10 μg/ml), human EGF (20 ng/ml) and hydrocortisone (0.5 μg/ml) (all supplements from Sigma).

### siRNA transfection

The effect of telomerase subunits downregulation (as well as cytotoxicity of transfection reagent and siRNA) was tested using the SRB assay as previously described [14] and compared with untreated control cells (data not shown). As a nontoxic concentration of transfection reagent, 1–2 μl of Lipofectamine2000 (Invitrogen, CA, USA) and 10–375 nM siRNA were chosen. Cells were cultured in DMEM medium, supplemented with 10 % fetal bovine serum without antibiotics. 24 h after seeding in 12-well plates the culture medium was replaced by OPTIMEM (without serum and antibiotics) followed by transfection with specific pooled siRNA (*TERT* from Dharmacon, ThermoFisher Scientific, IL, USA; *TERC* and *TP1* from Santa Cruz Biotechnology, CA, USA). Cells were cultured for 6 h in transfection mix and fresh, full serum medium was added. The experiment was carried on up to 24, 48 and 72 h. Transfection efficiency was confirmed using qPCR relative to FITC labeled, mock siRNA (Santa Cruz Biotechnology, CA, USA).

### Quantitative assessment of genes expression

#### Assessment of individual genes expression

After individual time intervals (24, 48, 72 h), quantitative analysis of genes expression was assessed as described previously [15] using qPCR. Briefly, total RNA was extracted with TriPure (Roche Diagnostics, IN, USA) [16]. cDNA was synthesized with Transcriptor First Strand cDNA synthesis kits (Roche Diagnostics, IN, USA), using 0.5 μg of total RNA and oligo dT primers. The real-time polymerase chain reaction for individual genes expression analysis (*TERT*, *TERC*, *TP1*) was carried out using LightCycler 2.0 with specific primers: TERTF, 5′-GCCGATTGTGAACATGGACT-3′; TERTR, 5′-CACCCTCGAGGTGAGACG-3′; TERCF, 5′-CGAGGTTCAGGCCTTTCA-3′; TERCR 5′-CCACAGCTCAGGGAATCG-3′; TP1F, 5′-GGGAGAGACCCAGTATGCAG-3′; TP1R, 5′-GCTGCAGGGTGGAGTTAGC-3′ designed with Universal Probe Library software (Roche Diagnostics, IN, USA). Amplification products of individual genes transcripts were detected via intercalation of the fluorescent dye SYBR Green (LightCycler FastStart DNA Master SYBR Green 1 kit, Roche Diagnostics, IN, USA). Cycling conditions for all amplicons were as follows: initially 95 °C for 10 min, followed by 45 cycles at 94 °C for 25 s, 60 °C for 25 s, and 72 °C for 15 s. All cycling reactions were performed in the presence of 2.5 mM MgCl_2_. Gene specific products were confirmed by melting curve analysis. The expression was normalized by the expression of the housekeeping gene GAPDH:GAPDHF, 5′-TTCGTCATGGGTGTGAACC-3′; GAPDHR, 5′-GATGATGTTCTGGAGAGCCC-3′.

#### Panel assays

The effect of TERT subunit downregulation on expression of genes contributing to apoptosis pathways was assessed using Real Time ready Human Apoptosis Panel, 96 (LightCycler 480 instrument; Roche Diagnostics, IN, USA) that enables profiling of 84 target genes and 7 housekeeping, reference genes (*ACTB*, *β2M*, *GAPDH*, *HPRT1*, *RPL13A*, *18S* and *YWHAZ*). The system is based on highly specific hydrolysis probes (Universal Probe Library). The assay was performed 72 h after transfection. The cDNA was prepared as described above.

### Telomerase activity assay

The effect of telomerase downregulation in breast cells was assessed using the quantitative TeloTAGGG Telomerase PCR ELISAPLUS kit (Roche Diagnostics, IN, USA), a modified original telomeric repeat amplification protocol (TRAP), [17] as previously described [18]. Briefly, a cell extract was prepared from MCF7 and MDA-MB-231 cells treated with specific siRNAs according to manufacturer’s protocol. For each assay, 2 μl of cell extract (corresponding to 2000 cells or 1 μg protein extract) was used. After 30 min incubation at 25 °C for primer extension, the PCR cycling conditions were: 94 °C for 5 min followed by 25 cycles at 94 °C for 30 s, 50 °C for 30 s and 72 °C for 90 s with final step at 72 °C for 10 min. The PCR products derived from telomerase elongation and internal standard were exposed to electrophoresis in a 12 % polyacrylamide gel (PAGE) followed by ethidium bromide staining and densitometric analysis (LabWorks System). Alternatively the results were quantitated using an ELISA assay and measurement of the absorbance at A450 nm against blank reference at A690 nm (Labsystems Multiscan RC; Helsinki, Finland). Heat-inactivated cell extracts and lysis buffer were also tested as a negative control. The HeLa cells extract (“low activity” standard provided by the supplier) was used as a positive control. Since only *TERT* downregulation revealed a significant telomerase inhibition, only *TERT* targeting siRNA was used in further experiments.

### Immunodetection

Cells were treated with anti-TERT siRNA and total protein was isolated using RIPA buffer (Sigma-Aldrich, USA). Samples containing 50 μg of protein were separated on a 7.5 % sodium dodecyl sulfate/polyacrylamide gel, and transferred onto a nitrocellulose membrane. The transfer was followed by blocking the membrane with 5 % skimmed milk in PBS-T. Rabbit antibodies directed against human *TERT* (Rockland, PA, USA) and GAPDH (Santa Cruz Biotechnology, CA, USA), which identify the proteins as single bands of 127 and 36 kDa, respectively, were added. After removal of the antibodies, anti-rabbit IgG secondary antibodies (Santa Cruz Biotechnology, CA, USA), labeled with horseradish peroxidase, were added and after 1 h incubation and washing, the protein was visualized on an X-ray film, using an enhanced chemiluminescence detection system (Roche Diagnostics, IN, USA).

### Cell cycle analysis: propidium iodide staining

Cell cycle analysis and apoptosis detection was performed as previously described [19]. Briefly, after treatment with individual siRNAs targeting *TERT* and *TERC* subunits, cells were washed with 1 ml of PBS and fixed with 70 % ethanol overnight at −20 °C. The ethanol was added dropwise to the cell pellet while vortexing to ensure fixation of all cells and minimize clumping. After washing twice in PBS, cells were centrifuged at 300 g and 250 μl of a solution containing 5 mg/ml propidium iodide, 1 μg/ml RNAse (Sigma, St Louis, MO, USA) in PBS was added to the pellet and incubated for 30 min at 37 °C in the dark. The samples were then analyzed by flow cytometry (Becton–Dickinson, FACScan).

### Apoptosis assessment: TUNEL assay

In order to estimate the influence of telomerase subunits downregulation on apoptosis in breast cancer cells a DNA fragmentation assay (In Situ Cell Death Detection Kit, TUNEL; Roche Diagnostics, IN, USA) was performed as previously described [18]. Briefly, after transfection with siRNA [100 nM], cells were detached with a 0.5 % trypsin–EDTA solution and collected. The cell suspension was fixed with 4 % paraformaldehyde and permeabilized with 0.1 % sodium citrate. After the reaction mixture was added, cells were incubated for 30 min and samples were analyzed by flow cytometry (Becton–Dickinson FACScanTM; Beckton Dickinson, Franklin Lakes, NJ, USA).

### Apoptosis assessment: annexin V and propidium iodide labeling

Annexin V detection was performed using FITC Annexin V Apoptosis Detection Kit I (Beckton Dickinson, Franklin Lakes, NJ, USA) according to manufacturer’s instructions. Briefly, MCF7 and MDA-MB-231 cells were treated with TERT siRNA (100 nmol) for 72 h and after collecting they were washed twice with cold PBS and then resuspended in 1× Binding Buffer. Cells were then transferred (100 μl of the solution (1 × 105 cells)) to a 5 ml culture tube followed by application of 2 μl of FITC Annexin V and 2 μl propidium iodide. Samples were gently mixed and incubated for 15 min at RT (25 °C) in the dark. Finally, 200 μl of 1× Binding Buffer was added to each tube and samples were analyzed by flow cytometry (Becton–Dickinson FACScanTM; Beckton Dickinson, Franklin Lakes, NJ, USA).

### Statistical analysis

All results are means from 3 and 7 separate experiments in triplicate. Statistical analysis was performed by one-way ANOVA followed by Tukey’s post hoc test. We used a *p* < 0.05 as the cutoff for significant difference.

## Results

### Telomerase subunits downregulation

The optimization study of transfection reagent and siRNA toxicity revealed the range of concentrations that did not show significant changes in cell survival (SRB analysis, data not shown). The concentration range (10–375 nM) and optimal ratio of Lipofectamine2000 and siRNA was established (3:1) and was consequently used in further experiments provoking up to over 70 % decrease in specific mRNA (*TERT*, *TR* and *TP1*) content. The optimal quantity of siRNA was chosen i.e. 100 nM and confirmed by qPCR (up to 90 % silencing, data not shown).

### Telomerase activity

**Fig. 1:**
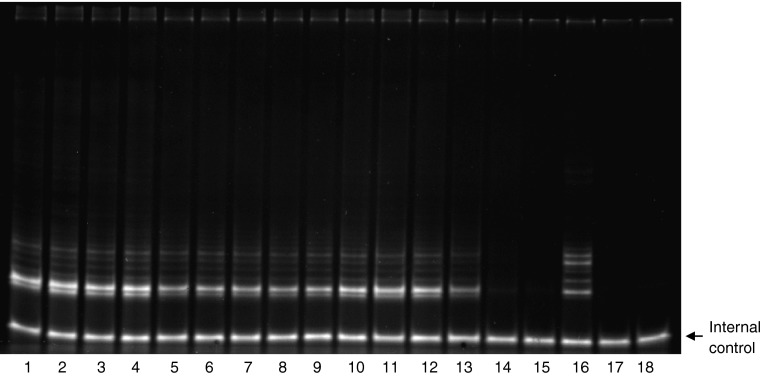
Telomerase activity in breast cancer (MCF7 and MDA-MB-231) and non-cancer (MCF10A) cells. (Typical TRAP-PAGE experiment) MCF7, MDA-MB-231 breast cancer and MCF10A non-cancer breast epithelial cells were subjected to telomerase activity assessment using a TRAP assay. For the analysis 0.2 μg of total protein extract was taken from breast cancer cells lysates. In order to detect the enzyme activity in MCF10A cells different quantities of total lysates were taken (up to 1.5 μg) due to a very low basal telomerase expression. The mean values of three independent experiments in duplicates were taken for significance calculation. Transfection reagent treated cells were used as control (100 %). The picture presents typical result out of seven separate experiments in duplicate. *p < 0.05. *1*,*2*–MCF7 control cells; *3*,*4*–carrier i.e. transfection reagent (Lipofectamine2000); *5*,*6*–TERT siRNA in MCF7 cells [100 nM]; *7*,*8*–TERT siRNA in MDA-MB-231 cells [100 nM]; *9*,*10*–mock siRNA [375 nM]; *11*,*12*–mock siRNA [100 nM]; *13*–HeLa cells extract (low standard); *14*,*15*–MCF10A extracts (1.5 and 0.2 μg); *16* HeLa cells extract (low standard) replication; *17* lysis buffer; *18* negative sample containing no lysis buffer; Internal control, internal standard to monitor PCR inhibition in every sample/lane

To confirm the specificity of telomerase downregulation an analysis of the enzyme activity was performed. It was analysed by PAGE (Fig. 1) and compared with the detection of PCR products by an ELISA assay (data not shown). In both assays similar results were obtained. The analysis of telomerase activity after transfection revealed that in MCF7 cells, TERT targetting siRNA caused more than 50 % enzyme inhibition after 72 h (results corresponding with Western blot analysis, data not shown). Targeting the same telomerase subunit gene in MDA-MB-231 cells resulted in 80 % inhibition of the enzyme activity compared to untreated control cells. This corresponded also with Western blot analysis. Alternatively, targeting the *TERC* or *TP1* subunits reveal slight and nonsignificant inhibition of the enzyme (data not shown). It was also impossible to achieve positive TRAP results in MCF10A noncancer cells (Fig. 1). Since targeting *TERC* and *TP1* subunits did not result in a significant telomerase activity inhibition further experiments were performed using anti-*TERT* siRNA only.

### Immunodetection

**Fig. 2:**
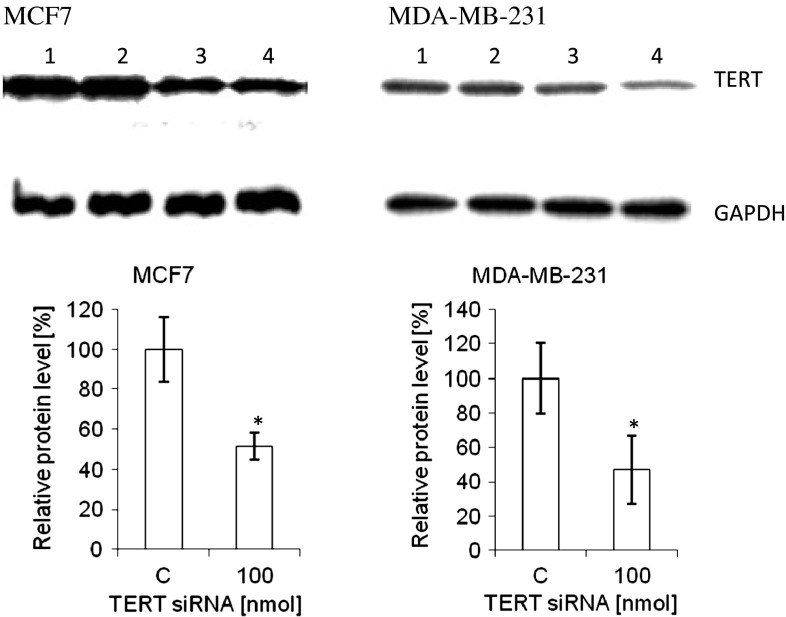
Efficiency of TERT downregulation at the protein level MCF7 and MDA-MB-231 cells were treated with 100 nM TERT siRNA (*lanes* 3 and 4) and after 72 h Western blot analysis was performed using a polyclonal antibody (Rockland, USA, PA). C, control cells, treated with transfection reagent (Lipofectamine2000; *lanes* 1 and 2); 100, cells treated with TERT siRNA [100 nM]. The picture presents typical result out of three separate experiments in duplicate. *p < 0.05

The effect of *TERT* downregulation after 72 h of transfection was studied at the protein level (Fig. 2). The downregulation of this subunit in MCF7 cells was corresponding to the results obtained at mRNA accumulation and telomerase activity analyses, showing about 50 % decrease in protein content. In MDA-MB-231 cells the effect was more efficient (over 50 % decrease) and also significant (*p* < 0.05).

### Cell cycle

**Fig. 3:**
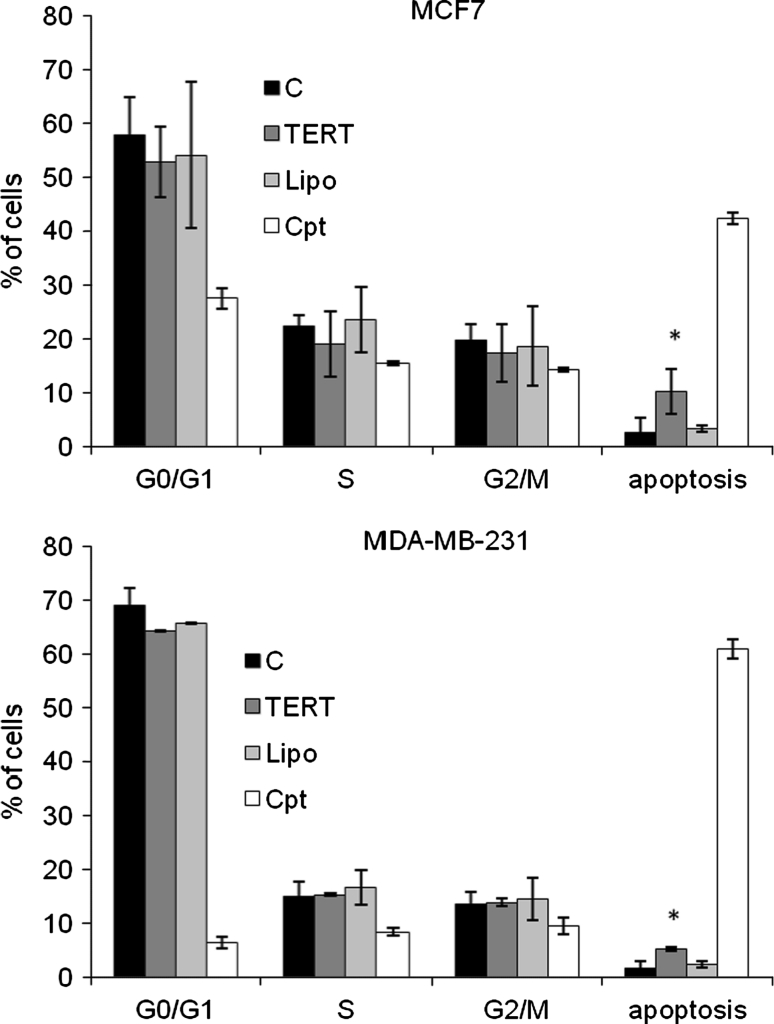
The effect of telomerase downregulation on the cell cycle of breast cancer cells Cells were treated with 100 nM specific siRNA or with camptothecin (as positive control) followed by propidium iodide labeling and flow cytometry analysis. The results were shown as relative apoptotic cell number. All experiments (three separate) were performed in triplicates, and *error bars* show the (±SEM) values. *p < 0.05. *C* control cells, *TERT* TERT targeting siRNA, *Lipo* transfection reagent (Lipofectamine2000), *Cpt* camptothecin [50 nM], *p < 0.05 (relative to control cells)

In order to analyse the influence of telomerase downregulation and inhibition on the cell cycle, a flow cytometry analysis using propidium iodide was performed. Transfection of cells with *TERT*-targeting siRNA provoked in both cell lines a significant increase in the number of apoptotic cells (*p* < 0.05). The apoptotic cell number in MCF7 cells was over 10 % versus 3.2 % in control cells. In MDA-MB-231 the apoptotic cell number was above 5 % relative to 2 % in control cells (transfection reagent treated) (Fig. 3).

### Apoptosis assessment

**Fig. 4:**
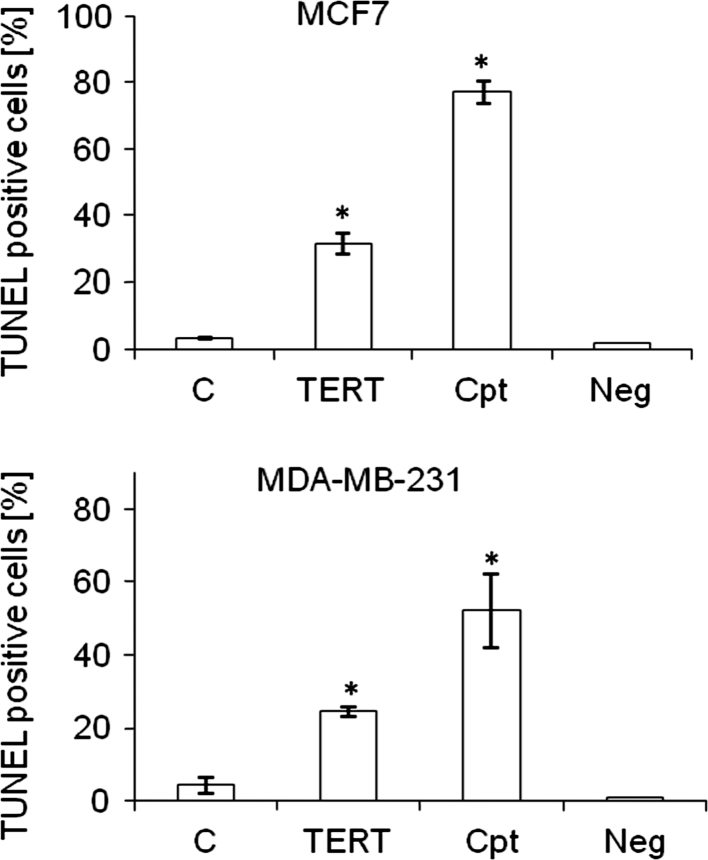
The effect of telomerase downregulation on DNA fragmentation Cells were treated with 100 nM specific siRNA or with camptothecin (as positive control) and subjected to TUNEL analysis using terminal transferase and flow cytometry analysis. All experiments (three separate) were performed in triplicates, and *error bars* show the (±SEM) values. *p < 0.05. *C* control cells treated with transfection reagent (Lipofectamine2000), *TERT* TERT targeting siRNA, *Cpt* camptothecin [50 nM], *Neg* negative sample, without terminal transferase; *p < 0.05 (relative to control cells)

**Fig. 5:**
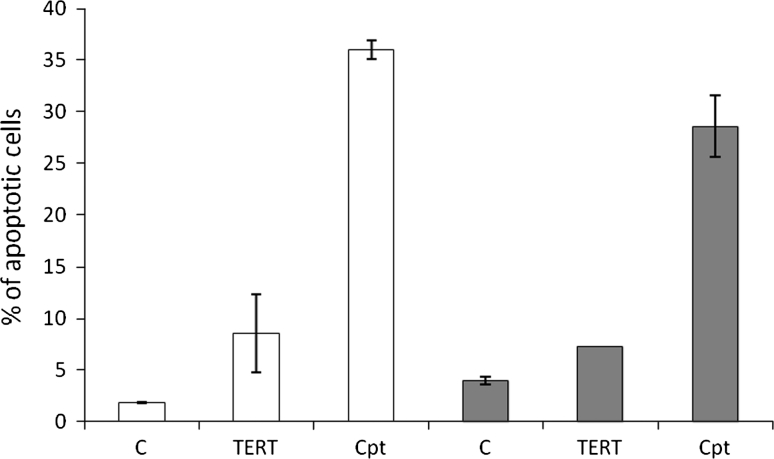
The effect of telomerase downregulation on apoptosis in breast cancer MCF7 and MDA-MB-231 cells MCF7 (*open bars*) and MDA-MB-231 (*grey bars*) were treated with 100 nM specific siRNA or with camptothecin (as positive control) and subjected to Annexin V and propidium iodide labeling followed by flow cytometry analysis. All experiments (three separate) were performed in triplicates, and *error bars* show the (±SEM) values. *p < 0.05. *C* control cells treated with transfection reagent (Lipofectamine2000), *TERT* TERT targeting siRNA, *Cpt* camptothecin [50 nM]; *p < 0.05 (relative to control cells)

To investigate the contribution of *TERT* downregulation to apoptosis, DNA fragmentation was studied in breast cancer cells after 72 h of treatment with TERT siRNA [100 nM]. *TERT* subunit downregulation resulted in a significant increase in TUNEL positive cells. In MCF7 cells TERT silencing caused about 30 % increase (*p* < 0.05) in DNA fragmentation relative to transfection reagent-treated control cells (3.1 %) (Fig. 4). Camptothecin, used as a positive control, provoked DNA fragmentation in almost 80 % of the cells. In MDA-MB-231 cells the DNA fragmentation signal was observed at the level of almost 25 % (*p* < 0.05) while control cells showed signal at 4.2 % (over 50 % in camptothecin treated cells). These results were consistent with the data obtained in the Annexin V detection assay. It was shown that TERT siRNA treatment of MCF7 cells provoked a significant increase of apoptotic cells up to almost 9 % comparing to almost 2 % in control cells (Fig. 5). In MDA-MB-231 cells we observed almost double number of apoptotic cells (over 7 vs below 4 % in control samples) provoked by *TERT* silencing. The results observed in TUNEL assay and PI staining as well as Annexin V labeling were positively correlated and the discrepancies (higher signal in TUNEL assay) might be provoked by the fact that in TUNEL assay cells in early stages of apoptosis up to the stage when the morphological changes occur are stained.

Moreover, it is interesting to note that the percentage of TUNEL (+) cells only significantly correlated with double positive cells [Annexin-V (+)/PI (+)], but not with only Annexin-V (+) cells (Fig. 3). This can be explained by the common understanding that DNA fragmentation usually occurs at the late stage of apoptosis caused by endonuclease activation (Arends et al. 1990). Such correlations also suggest that the double positive cells [Annexin-V (+)/PI (+)] are more likely to be late apoptotic cells, rather than necrotic cells. Results from this study thus indicate that both Annexin-V staining for the identification of PS exposure and the TUNEL assay for the measurement of DNA fragmentation are valid tools for the assay of apoptosis in sperm.

### Influence of *TERT* downregulation on apoptosis engaged genes expression

**Fig. 6:**
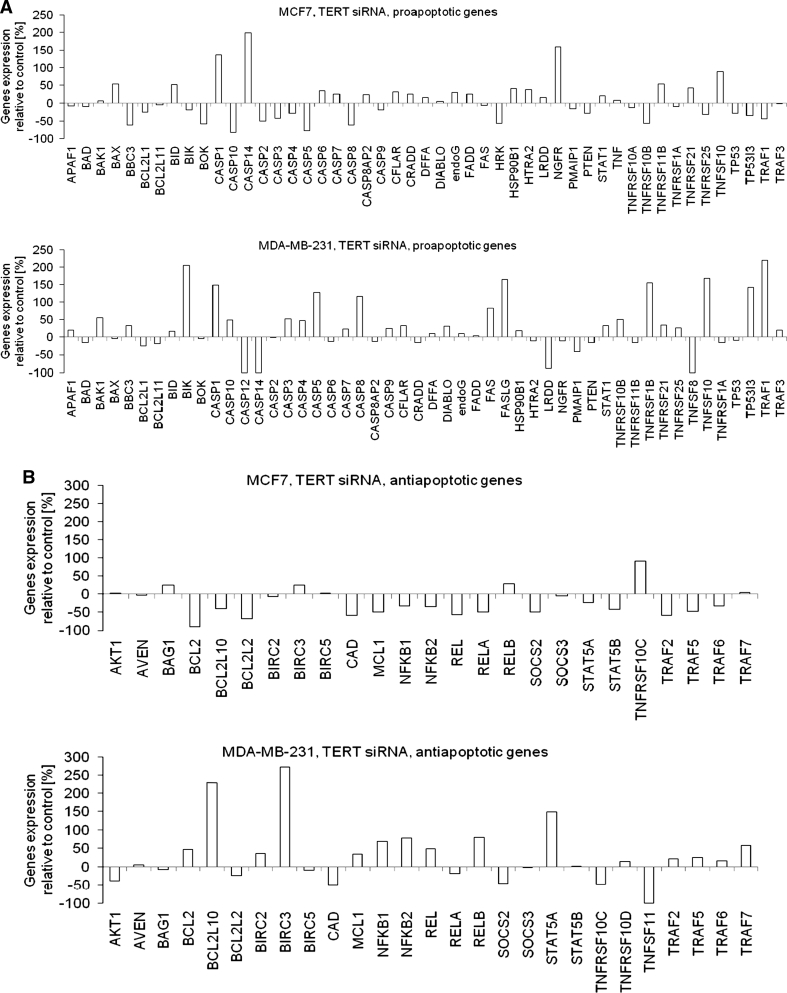
The effect of telomerase silencing on expression of genes contributing to apoptosis control Cells were treated with 100 nM of TERT targeting siRNA and after 72 h the expression profile of proapoptotic (**a**) and antiapoptotic (**b**) genes in MCF7 and MDA-MB-231 cancer cells was assessed using qPCR. The experiment was performed in duplicates. The graphs represent the increase or decrease of the expression of the indicated genes relative to untreated control cells, (baseline at “0″ level)

The analysis of the panel of genes that contribute to apoptosis demonstrated that expression of numerous proapoptotic genes was induced (Fig. 6a). These were e.g. *caspase-1* and *caspase-14* genes, *NGFR* and *TNFSF10* which were induced by about two times and more in MCF7 cells relative to controls. However, there were also numerous genes from this group that were repressed compared to controls, e.g. caspases 5, 8, 10 and *BOK*.

The analysis of genes expression in MDA-MB-231 cells revealed that two of them were extremely highly induced, i.e. *TNF* and *TNFRSF10A* (20 and 13 fold increase, respectively). There was a group of proapoptotic genes that were also induced, however not so spectacularly, e.g. *BIK* and *TRAF1* (both induced by more than 200 %). Caspases 1, 5, 8, *FASLG*, *TNFRSF1B* and *TNFSF10* were also significantly upregulated (all about twofold, relative to control cells) (*p* < 0.05).

In the analysis of antiapoptotic genes (Fig. 6b) it was demonstrated that in both cell lines most of the genes were repressed. In MCF7 cells these were e.g. *BCL2* (almost 90 % decrease), *BCL2L2* (almost 70 % decrease) and *TRAF2* (almost 60 % decrease). There were also some genes that were simultaneously induced i.e. *TNFRSF10C* (over 90 % increase) and *RELB* (about 30 % increase). When MDA-MB-231 cells were analysed, we observed induction of *BCL2L2* or *BIRC3* (both induced by about twofold relative to control cells). Downregulation of only a few antiapoptotic genes was accompanying *TERT* downregulation in MDA-MB-231 cells e.g. *CAD* (50 % decrease) or *TNFSF11* (totally downregulated to an undetectable level).

## Discussion

Downregulation of telomerase in combination with doxorubicin in breast cancer cells was shown to potentiate the cytotoxic effect of the drug [20]. We went further, and similarly to the study on Barrett’s adenocarcinoma cells [21] we show detailed consequences of *TERT* downregulation. We show that in breast cancer cells, *TERT* downregulation with low doses of specific siRNA induces apoptosis. This effect is accompanied by significant DNA fragmentation, accumulation of apoptotic cells and induction of numerous genes regulation engaged of apoptosis. We report here that targeting *TERT* can down-regulate telomerase activity and inhibit proliferation of breast cancer cells in vitro, suggesting the potential for therapy.

It was suggested that apoptosis of cancer cells is caused mainly by from a decrease in telomere length [21]. However, it is known that these two factors do not have to be always associated. We show that cell death may appear much faster than telomere length erosion and is detected just after 72 h after *TERT* downregulation (is really telomere shortening shown?). This implicates that this process is telomere length independent. We also postulate that the effect of telomerase elimination is cell type dependent. One of the genes that were modulated at a significant level after *TERT* silencing was *Bik*. Interestingly, it was slightly decreased in MCF7 (estrogen dependent) but significantly increased by about twofold in MDA-MB-231 (estrogen independent) cells. However, it is known that the function of this protein is strongly correlated with estrogen receptor signaling pathway (ER represses Bik expression; [22]). Additionally, another important factor contributing to the proapoptotic activity of Bik are changes in the expression of the antiapoptotic proteins Bcl-2 (decreased in MCF7 and increased in MDA-MB-231 cells) and Bcl-XL which are Bik target proteins strongly affecting the efficiency of the Bik-induced apoptosis [23]. Another gene that was significantly induced after *TERT* silencing was *caspase-1* (in both cell lines), which is a member of the inflammatory caspase family which activates proIL-1β and proIL-18. Similarly to studies in SEG-1 Barrett’s adenocarcinoma cells [21] we observed that telomerase suppression in MDA-MB-231 cells was also associated with up-regulation of genes for FasL, Fas and caspase-8. This suggests that in these cells telomerase downregulation leads to activation of death receptor-mediated apoptosis. We also found upregulation of genes for caspase-7 and caspase-3 (in MDA-MB-231 cells) which are executioner caspases implicated in the cleavage of PARP in the final stages of apoptosis. We also observed induction of nerve growth factor receptor in MCF7, while it remained unchanged in MDA-MB-231 cells. Noteworthy, it belongs to TNFR domain proteins and DEATH domain proteins and is related to estrogen receptor signaling [24]. The other proapoptotic gene Apo-2L (TNFSF10, tumor necrosis factor/ligand superfamily, member 10) was also significantly induced in both cell lines. The TP53I3-tumor protein p53-inducible protein 3 (p53 pathway element) was significantly induced only in estrogen independent (but p53 mutant) MDA-MB-231 cells after 72 h. Noteworthy, upon identification of the telomere-capping shelterin complex, p53 has been established as a downstream effector of the DNA damage signaling emerging from a specific shelterin component at uncapped telomeres [25].

Among the antiapoptotic genes we also found some that were significantly modulated at the mRNA expression level. First, *Bcl*-2 which is controlled by ER status [26, 27]. It was significantly reduced in MCF7 while in MDA-MB-231 it was initially induced (24 h) and finally (72 h) restored to the basal level. This, however, might be related to some primary nonspecific effects (this effects should be mentioned). Similar results were obtained in prostate cancer cells transfected with anti-TERT siRNA, which caused significant inhibition of enzyme activity (up to 80 %). This led to a marked reduction of TERT mRNA and protein expression, followed by a significant inhibition of cell proliferation within a few days [28]. Other studies revealed that using retroviral delivery of siRNAs specific for *TERT*, successfully inhibited telomerase activity in cervical cancer cell lines.

These findings suggest that a siRNA-based strategy can be applied to the development of novel telomerase inhibitors, the antitumor effects of which may be enhanced in combination with ionizing radiation and chemotherapy [29, 30]. A significant reduction in telomere length was also observed in HT29 cells after telomerase downregulation which was followed by limited proliferation [31]. Anti-*TERT* siRNA was also reported to inhibit the proliferation of hepatocarcinoma (HepG2) cells [32].

The cells we studied (MDA-MB-231 and MCF-7) differ in ER or PGR status as well as caspase-3 activity, p53 status and many more factors. Thus, our understanding of the mechanisms controlling telomerase and estrogen-dependent cell fate requires further detailed studies. The gene expression studies provide only an overview of changes in genes involved in the apoptotic pathways. Although the expression changes were followed by apoptosis and DNA fragmentation, further studies of changes in protein expression and activities would be required to fully define the signaling pathways. Especially since the observed apoptosis levels measured by DNA fragmentation or by flow cytometry gave results that did not correlate completely. This suggested a complexity of mechanisms that take place in different stages of cell death pathway. Additionally, further studies in telomerase expressing normal cells are needed to determine whether effects of the anti-telomerase siRNA are selective for cancer cells.

### Potential clinical translation

There are some discrepancies concerning the question about the telomerase subunit that should be targeted in order to “turn off” the whole complex. As we demonstrated in breast cancer cells, *TERT* downregulation seems to be the most efficient. However, probably an even more effective gene therapy could be achieved by a combination of *TERT* with other subunits. On the other hand this might also depend on the siRNA sequence and specificity, as well as transfection method. One should also not forget about the contribution of individual telomerase subunits in other metabolic processes and potential side effects. Some researchers suggested that strategies for telomerase inhibition that require downregulation of TERT mRNA may be less straightforward than those that targeting TR [33]. Some other reports demonstrated that anti-*TERT* siRNA can cause effective suppression of telomerase and to apoptosis in A549 lung adenocarcinoma cells. *TERT* siRNA may, therefore, be a strong candidate for highly selective therapy for chemoprevention and treatment of lung adenocarcinoma [34]. Interestingly, to confirm this hypothesis, we must remember that patients with high telomerase expression responded poorly to chemotherapy in terms of disease-free and overall survival, but fared better if treated with endocrine therapy [9].

TERT mRNA expression significantly correlates with telomerase activity in human breast cancer. This is consistent with the hypothesis that TERT is the catalytic and rate-limiting determinant subunit of the enzyme [35]. Altogether, our data show that blocking telomerase activity can be used as an effective way to induce death of breast cancer cells. We analyzed the telomere length (according to a protocol previously described in [36 **]** and we observed that TERT siRNA treatment did not provoke significant telomere length change after 72 h of exposure (however, slight but not significant shortening was observed, data not shown). Thus, it may be that treatment of cancer patients with telomerase inhibitors does not have to be a long-term treatment and does not have to depend directly on replicative senescence and telomere length. It looks that only *TERT* downregulation starts all the long pathway to induce apoptosis and cancer cell elimination. This however makes high demands on the profile of side effects of this approach especially since not only tumor cells but also stem cells and germ cells also express telomerase activity. However, as these cells usually have, longer telomeres, lower telomerase activity and the proliferation rates are in general lower compared with tumor cells [37]. Thus, the effects of treatment of these cells should be moderate. On the other hand, the complex regulation of telomerase activity and expression [38, 39] strongly suggests that telomerase may play roles other than in cancer and aging, such as in the Wnt signaling pathway and in RNA processing [40]. This implicates the need for careful and thoughtful conclusions concerning telomerase modulation and potential side effects. However, so far, there are no data to suggest stopping trials on the run.
